# Particulate debris from a titanium metal prosthesis induces genomic instability in primary human fibroblast cells

**DOI:** 10.1038/sj.bjc.6600758

**Published:** 2003-02-18

**Authors:** N Coen, M A Kadhim, E G Wright, C P Case, C E Mothersill

**Affiliations:** 1 Radiation and Environmental Science Centre, Dublin Institute of Technology, Kevin St, Dublin 8, Ireland; 2 Radiation and Genome Stability Unit, Medical Research Council, Harwell, Didcot, Oxon OX11 ORD, UK; 3 Department of Molecular and Cellular Pathology, Ninewells Hospital and Medical School, University of Dundee, Dundee, DD1 9SY, UK; 4 Avon Orthopaedic Centre, Southmead Hospital, Bristol BS10 5NB, UK

**Keywords:** hip implants, genomic instability, chromosome aberrations, delayed reproductive death

## Abstract

Previous studies detected both lethal and cumulative chromosomal aberrations in bone marrow and peripheral blood of patients with worn hip and knee replacements. This study shows that wear debris from a worn titanium metal on high-density polyethylene hip replacement also produces chromosomal instability and reproductive failure in cell culture. The progeny of these treated cells also displayed chromosomal instability, mainly consisting of chromatid breaks and minutes, and reproductive failure as determined by clonogenic survival many generations postexposure. These delayed effects are similar to those caused by the heavy metals cadmium and nickel and to those seen for low-dose radiation. These findings may have important implications with regard to the long-term risks of joint replacement surgery. This highlights the need for long-term epidemiological studies of patients with surgical implants.

Prosthetic joint replacement is a common surgical procedure with at least 1 million hip prosthesis being implanted each year, worldwide (Bullough, 1994). Prosthetic joint replacements are typically trouble free; however, approximately 20% fail within 10 years (Saeed and Revell, 2001). The expectations for this procedure continue to rise as it is being extended to younger and more active patients whose life expectancy exceeds 30 years postsurgery (Jacobs *et al*, 1998). These metal prostheses undergo corrosion in the body (Merritt and Brown, 1996; Jacobs, 1999). With the passage of time, this can result in the generation of particulate debris that can accumulate in the tissues adjacent to the prosthesis (Case *et al*, 1994; Jacobs *et al*, 1996). Particulate debris has also been shown to accumulate in the lymph nodes, liver, spleen and bone marrow (Langkamer *et al*, 1993; Case *et al*, 1994; Jacobs, 1998), and subsequently may elicit important biological responses. Inflammatory responses have been shown to be initiated by particulate debris and they have been implicated in the induction of osteolysis through macrophage activation (Maloney *et al*, 1993; Jacobs *et al*, 1998). This appears to play a role in implant failure (Breen and Stoker, 1993). These studies indicate that particulate debris is not biologically inert. Owing to its of its superior mechanical properties and biocompatibility, titanium is widely used for joint prostheses. Titanium alloys ‘*in vivo*’ form a stable oxide layer, which greatly enhances the inertness of titanium. However, because of mechanical and biological disturbance the destabilisation of this oxide layer is inevitable leading to the release of titanium ions and particulate debris (Breen and Stoker, 1993).

Past studies have placed their attention on prosthetic loosening, implant failure and inflammatory response. They have ignored the consideration of the long-term consequences of debris on cellular viability and the induction of genomic instability. Genomic instability is a phenomenon and has definitively been shown to occur postirradiation (Kadhim *et al*, 1992; O'Reilly *et al*, 1994; Seymour *et al*, 1996). It has also been shown by our group to be induced by heavy metals and other environmental toxins (Mothersill *et al*, 1998; Coen *et al*, 2001). Genomic instability can manifest itself in many ways including lethal mutation/delayed reproductive death (Seymour *et al*, 1986; Mothersill *et al*, 1995; Mothersill *et al*, 1998), delayed appearance of nonclonal chromosomal aberrations in progeny of exposed cells (Kadhim *et al*, 1992,^1995^) and delayed apoptosis (Lyng *et al*, 1996). These are regarded as delayed effects resulting from transmission of genomic instability to the progeny of treated cells. Characteristically, it is induced at frequencies greater than that of natural spontaneous mutations (Kadhim *et al*, 1996). Once induced, genomic instability is effectively permanent and has been shown to persist *in vitro* and *in vivo* for many cell doublings (Seymour *et al*, 1986; O'Reilly *et al*, 1994; Watson *et al*, 1996). The phenomenon of aneuploidy and its relevance to the genetic instability of cancer cells has recently been documented (Duesberg *et al*, 1998). In a study by Doherty *et al* (2001), a five-fold increase in aneuploidy was observed in patients with titanium–vanadium–aluminium (TiVaAl) prosthesis at revision surgery compared with those at primary arthroplasty. While the induction of genomic instability by environmentally relevant concentrations of metals has been documented, its induction by metal devices has not been studied.

The generation of particles from a metal prostheses, which considerably increases the surface area exposed, favours the release of ions that could influence cellular response of the patient to the implant (Merritt and Brown, 1996; Jacobs *et al*, 1998). This paper investigates the induction of genomic instability and tetraploidy by particulate titanium debris generated from a metal prosthesis. This study may highlight that at revision surgery although the stimulus may be removed the effect can persist. As there are many concerns about the adverse biological responses that could occur during the long term in patients with metal prosthesis, there is the need for more extensive cohort studies to be established. The end points of genomic instability used in this study were lethal mutations/delayed reproductive death and delayed cytogenetic abnormalities in the progeny of cells that were exposed to the debris.

## MATERIALS AND METHODS

### Cell culture

The cells used in this study were early passage, normal human fibroblasts (HF19 cells). These cells have been well characterised for the cytogenetic and survival end points used in this study and have the advantage of a normal karyotype (Kadhim *et al*, 1997). For these experiments, passage 4 cells were removed from liquid nitrogen storage and resuspended in 3 ml minimum essential medium, alpha modified (Sigma Aldrich, UK), supplemented with 10% foetal calf serum (Sigma, Aldrich, UK), 2 mM L-glutamine, 50 IU ml^−1^ penicillin and 50 *μ*g ml^−1^ streptomycin. The cells were cultured in an atmosphere of 5% CO_2_ in a 24 cm^2^ tissue culture flask (Nunc; T24) at 37° until confluent. The cell number was then expanded to provide sufficient cells for all planned experiments. Passage 5 cells were used for all experiments.

### Titanium debris

Wear debris was extracted from periprosthetic tissue of a patient undergoing revision total hip arthroplasty. According to methods described by Doherty *et al* (2002), this tissue from a patient with TiVaAl prosthesis was digested in 1% trypsin-collagenase gently mixed and incubated at 37°C for 24 h. Wear debris was separated from digested tissue on histoplaque (Sigma, Aldrich, UK) by centrifuging for 25 min at 3500 r.p.m. The metal layer was removed, washed in PBS, and then resuspended in PBS and stored at −20°C. This was the stock solution and contained approximately 6.4 g of metal in 1 ml of PBS. From this dense solution of titanium debris a series of dilutions were made using dilution factors ranging from 1 : 50 to 1 : 100,000. These dilutions were made up in cell culture media. The particle suspensions were continually mixed to maintain uniform particle distribution at the time of dispersal onto cells.

### Cytogenetic analysis

5×10^5^ cells, plated in 75 cm^2^ tissue culture flasks, were allowed to attach for several hours. These were then exposed to 0, 1 : 50 000, 1 : 5000, and 1:500 dilution factor of titanium debris for a period of 24 h. The cells that received 0 dose of titanium debris represent control cells. After 24 h the cells were washed twice with PBS to remove all debris and 10 ml of fresh growth medium was added. After 48 h postexposure to the titanium debris (approximately three population doublings), cells were harvested for cytogenetic analysis to examine the initial effects caused by titanium debris. The delayed effects were assessed at passage 2 (approximately 10 population doublings postexposure). Chromosome preparations were made by accumulating metaphases in the presence of 0.05 *μ*g ml^−1^ colcemid for 2 h, followed by treatment with 0.5% (w v^−1^) potassium chloride and fixation in methanol : acetic acid (3 : 1 v v^−1^). Fixed cells were spread on to slides and air dried. These were then stained using 5% Giemsa. A minimum of 100 well-spread metaphases was scored per dose point to determine the frequency of aberrations.

### Clonogenic analysis

HF19 cells were plated in 24 cm^2^ flasks (Nunc, Denmark) at dilutions adjusted to yield approximately 100 viable colonies according to the method established by Puck and Marcus (1956). Cultures were established in 3 ml of growth medium and cells were allowed to attach. After attachment cultures were initiated with a range of titanium debris dilution factors, 0, 1 : 100 000, 1 : 50 000, 1 : 10 000, 1 : 5000, 1 : 1000, 1 : 500, 1 : 100, 1 : 50, for a period of 24 h. Six flasks of cells were exposed to each dilution, three of these were stained using carbol fuchsin (Ziehl Niehlson; 20%) to assess initial colony formation. The cells in the remaining flasks were grown and passaged to allow examination of effects in distant progeny according to the methods described by Seymour *et al* (1986). Delayed death was assayed at passage 2, approximately 15 population doublings postexposure to the debris.

### Statistical analysis

All experiments were repeated three times and within each experiment points were set up in triplicate. Results are expressed as the mean±s.e. Fisher's exact test was performed on the cytogenetic results where treatments were compared to controls.

## RESULTS

### Cytogenetic analysis

Chromosomal instability has been defined as the persistence of nonclonal chromosomal aberrations with a high frequency of chromatid-type aberrations in the clonal descendants of a single treated cell. It is evident from this study that the induction and transmission of chromosomal instability in the progeny of primary human fibroblasts occurs as a result of exposure to titanium debris (Table 1

**Table 1:** The cytogenetical analysis of HF19 cells postexposure to a range of dilutions containing titanium debris

**Dilution**	**Population doublings**	**No. of aberration**	**No. of unstable aberration**	**No. of other aberration**	**No. of tetraploid cells**	**Endoreduplication**
C	3	1	0	1	0	4
	15	2	1	1	3	0
						
1 : 50 000	3	7	3	4	7^a^	9
	15	9	5	4	5	0
						
1 : 5000	3	5	3	2	11^b^	7
	15	9^a^	7^a^	2	9	0
						
1 : 500	3	14^b^	8^b^	6	11^b^	5
	15	20^b^	10^a^	10^a^	10^a^	0

The results reflect aberrations out of 100 well-spread metaphases, which were scored for each treatment. There are high incidences of unstable aberrations, which include chromatid breaks, fragments and minutes, as compared to control. Other aberrations were also observed, including chromatid and chromosome gaps, chromosome breaks and acentrics. Endoreduplication and tetraploidy both showed increased incidences.

a *P*⩽0.05.

b *P*<0.005 as determined by the Fisher's exact test.

). In cultures treated with 1 : 50 000 and 1 : 5000 dilution factors of titanium debris, the induction of unstable aberrations was low compared to cultures treated with 1 : 500 dilution. This indicates a dose–response of the cells to increased concentrations of the particulate titanium debris. The most frequently encountered aberrations are of the unstable type (chromatid breaks, chromosome fragments and minutes), but other aberrations were also evident such as chromosome and chromatid gaps and chromosome breaks. These aberrations were evident after 15 population doublings, with a greater frequency of aberrations expressed in the progeny than in the initially treated cells. The cytogenetic analysis also demonstrated an increased expression of tetraploidy in all the various dilutions analysed. This expression of tetraploidy was most evident after exposure to 1 : 500 dilution factor of titanium debris. The induction of endoreduplication is also evident from the results (Table 1). Endoreduplication is where the cell undergoes two mitosis without cell division intervening (Aardema *et al*, 1998).

### Clonogenic analysis

Figure 1

**Figure 1:**
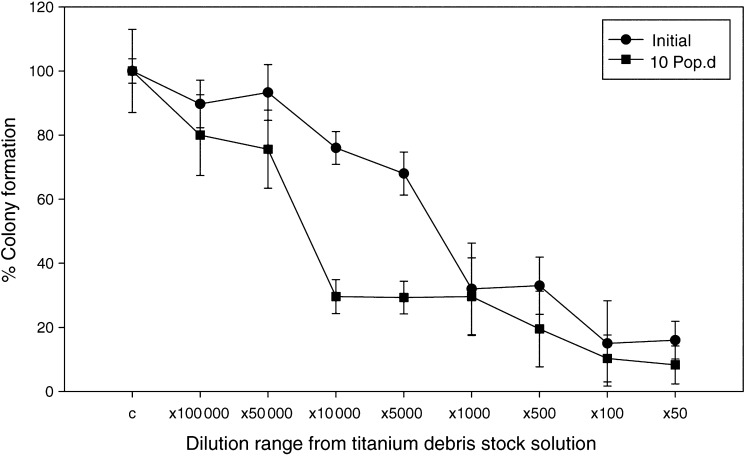
The initial survival curve reflects the percentage survival with respect to colony formation of HF19 cells exposed to titanium debris for 24 h. The percentage survival of the progeny of these cells (these cells were not treated) was examined 10 population doublings postexposure to titanium debris. All values were expressed as a percentage of control where control was expressed as 100%.

shows the initial toxicity of HF19 cells treated with titanium debris for 24 h. The most dilute concentrations of titanium debris, 1 : 100 000 and 1 : 50 000, have little effect on the reproductive integrity of the cells, reducing the cloning efficiency only by 10%. Concentrations greater than 1 : 50 000 cause a drop in clonogenic survival. The results for delayed reproductive death in the progeny of these cells correspond to 10 population doublings postexposure. These cells have not been treated with titanium debris but are the progeny of the initially treated cells. These data demonstrate that at the sublethal concentrations of titanium debris, determined from the initial toxicity, the reproductive survival is greatly reduced. At dilution factor 1 : 10 000, the progenitor population that received the titanium debris showed survival of 70% but the progeny of these cells express a reduced colony formation of less than 30% of that found for the untreated control progeny.

## DISCUSSION

It has been well established that some metals, which are used in metal implants, have carcinogenic potential to humans when exposure occurs in their ionic form and via other routes (IARC, 1987). It has yet to be established whether metals used in metal prosthesis in particulate form have themselves the capability to cause malignant transformation of cells. This paper provides direct evidence that titanium obtained from a metal prosthesis can induce characteristics that are associated with genomic instability in the progeny of treated primary human cells. The genomic instability demonstrated in this study is marked by a reduced clonogenic survival in the progeny of treated cells 10 population doublings postexposure and a persistent level of cytogenetic abnormalities.

The clonogenic analysis demonstrates lethal mutations/delayed reproductive death in the progeny of cells treated with titanium debris. This expression of damage is similar to earlier observations that a wide range of chemicals could reduce the reproductive integrity of progeny cells for many generations following the initial acute exposure (Mothersill *et al*, 1998; Coen *et al*, 2001). It was observed from the clonogenic analysis that the titanium debris dilutions, which produced no apparent cytotoxicity to the initially exposed cells, allowed the survival of populations of cells, which are more prone to exhibit reproductive failure during subsequent cell divisions. For example at 1 : 10 000 dilution factor of titanium debris, almost 70% of the cells survive to express the genomic instability phenotype compared with less than 30% after treatment with 1 : 100 dilution. At the 1 : 10 000 dilution factor the progeny shows over 50% reduction in survival compared to the initial acute toxicity, whereas 1 : 100 dilution only shows less than 10% reduction compared to initial acute toxicity. Cells appear quite normal initially and show no evidence of increased cytogenetic abnormalities or reproductive failure. However for several generations of division postexposure, they begin to show high levels of nonclonal cytogenetic aberrations and reproductive failure. Consequently, low-dose exposure may have important consequences. This has been observed for all agents tested so far which show inducible genomic instability. The mechanism of induction and transmission of these effects is unknown but persistent oxidative stress has been documented in murine haemopoietic cells (Clutton *et al*, 1996). The perpetuation of the observed insidious damage expressed in an ‘*in vivo*’ situation may be prompted by the induction of reactive oxygen species. Metal prosthesis may elicit an inflammatory response whereby macrophages accumulate at the site of injury (Black and Hastings, 1998). During phagocytosis the macrophages generate highly reactive oxygen metabolites. This increase in production of superoxide anion and hydroxyl radicals as well as released lysomal hydrolyses by activated macrophages may augment cellular damage (Anderson and Miller, 1984). These oxidative species may also oxidise titanium peroxy compounds that eventually degrade into titanium dioxide and hydrogen peroxide (Overgaard *et al*, 1998). Further decomposition of hydrogen peroxide and titanium oxide creates the highly detrimental hydroxyl radical and superoxide (Tengvall *et al*, 1989; Klinger *et al*, 1997).

It has been hypothesised by Mothersill *et al* (1996) that lethal mutations/delayed reproductive death represents a sort of ‘cleansing mechanism’ removing unrepaired damage in the genome. Evidence for this has been obtained by Mothersill *et al* (1999) who examined cells from genetically different mice, which were previously shown to have different susceptibilities to cytogenetic instability (Watson *et al*, 1997). This study revealed that in the susceptible mice there was a low level of both early and delayed cell death (necrosis and apoptosis) in exposed cultures following irradiation, but in the mice that were resistant to the induction of cytogenetic instability, there was a high initial and delayed level of cell death. This strongly suggests that the death end points of genomic instability are acting to remove damaged cells from the proliferative population. In this study low-dose exposure allows greater survival of cells to express damage, reflected by the low cloning efficiency in the progeny of these cells. Thus, the delayed reproductive death observed is a measure of damage induced by titanium debris. Failure to respond to this damage by death increases the probability that the unstable cell will survive and divide. These results are very similar to those ascertained by this group for chemical carcinogens and radiation (Mothersill *et al*, 1998; Coen *et al*, 2001).

From the cytogenetical analysis it can be observed that the frequency of aberrations is elevated compared to control, most of these are of the unstable type arising *de novo.* These values are lower compared to results obtained for radiation (Kadhim *et al*, 1997) but they show a definite trend towards genomic instability. Like radiation, heavy metals induce stress responses and proteins such as metallothionein are upregulated (Duesburg and Rasnick, 2000). These protect cells from damage by these insults by inducing repair pathways and eliminating individual cells by apoptosis. The lower frequencies of persistent damage in metal-treated cells compared to radiation-treated cells may relate to metallothionein proteins that specifically deal with metal toxins. The cytogenetical analysis shows a phenomenon that is not seen postexposure to radiation, which is the persistent induction of tetraploidy. It has been postulated that such ploidy changes destabilise the karyotype leading to a tumorigenic karyotype (Duesburg *et al*, 1998; Duesburg and Rasnick, 2000). It is also known that tetraploidy may lead to immortalisation of cells ‘*in vitro*’ and to transformation during the process of carcinogenesis (Duesburg and Rasnick, 2000). These observations lead us to speculate that although conditions ‘*in vitro*’ are different to those ‘*in vivo*’, particulate debris from metal prostheses might leave the patient susceptible to carcinogenic initiation. A two-fold increase in chromosomal translocations was observed in patients with metal on plastic total hip replacements undergoing revision arthroplasty compared with those at primary arthroplasty (Doherty *et al*, 2001). Higher rates of chromosomal aberrations were observed in local bone-marrow cells adjacent to the prosthesis at revision surgery compared with bone marrow from the iliac crest from the same patient (Case *et al*, 1996). These aberrations mainly consisted of chromatid breaks. In some cancer incidence studies, it has been observed that there were increased incidences of lymphomas and leukaemia in patients with prosthetic joint replacements, but there were decreased incidences of breast, colon and rectum cancer (Gillespie *et al*, 1988; Visuri and Koskenvao, 1991). These studies were followed up over a 10-year period postoperative procedure, which probably explains the significant increase in the short latency-type haematopoietic cancers. The latency period for particulate-induced solid tissue malignancy could take up to 20 years or more to manifest itself. Mechanical implantation or inhalation of metal dust may proceed to neoplastic transformation by a chemical route after corrosion or by aggregation of corrosion products around the implant or remote sites (Black and Hastings, 1998). Cytogenetic analysis also showed the induction of endoreduplication. This is where the cells undergo two DNA syntheses without cell division intervening (Aardema *et al*, 1998) and its occurrence may provide a mechanistic explanation for the induction of tetraploidy by titanium debris. High frequency of tetraploidy is associated with malignant melanoma and lymphoma (Satoh *et al*, 2000). Results from this paper revealed significant increases in the induction of tetraploidy in the fibroblast cell after a brief exposure to 1 in 500 dilution of the titanium debris.

In conclusion, this preliminary evaluation of particulate titanium debris from metal prosthesis has revealed that corrosion debris induce significant changes in treated cells to cause a measurable response. Increased levels of chromosomal aberrations and tetraploidy as well as lethal mutations/delayed reproductive death were observed in cells treated with titanium debris and their progeny. This damage was expressed for up to 10 population doublings postexposure for lethal mutations/delayed reproductive death and 15 population doublings postexposure for chromosomal instability. This study demonstrated that once the stimulus is removed the damage persists for many cell population doublings. In both animal and human studies, neoplastic transformation requires some time to pass between exposure (initiation) and manifestation of neoplastic transformation (Hennig *et al*, 1992). In humans, the latency period is typically 15–20 years and may be as long as 40 years (Black, 1999). Thus, the appearance of metal carcinogenesis in humans may be awaiting the passage of unelapsed latency period in the large number of younger patients who have received implants. This highlights the need for long-term epidemiological studies to determine the risks involved for young patients receiving surgical implants.
